# Corporate governance practices, barriers and drivers: A survey dataset

**DOI:** 10.1016/j.dib.2020.106603

**Published:** 2020-11-29

**Authors:** Muhammad Arslan, Ahmad Alqatan

**Affiliations:** a Bang College of Business, KIMEP University, Kazakhstan; b Bangor Business School, Bangor University, United Kingdom

**Keywords:** Corporate Governance, Survey questionnaire, Pakistan stock exchange, Factor analysis, Regression, CG Index, Emerging economy

## Abstract

The dataset presented in this paper is used to examine the corporate governance (CG) practices and compute the CG index among Pakistan Stock Exchange (PSX) listed firms. Dataset is useful in exploring the different barriers to and drivers of good CG practices. The survey questionnaire was distributed to 350 respondents from 350 PSX listed firms, nevertheless, 120 respondents returned the questionnaire. After reviewing the filled questionnaires, fifteen questionnaires were found to be incomplete. Therefore, the final sample consisted of 105 respondents from 105 PSX listed firms. The survey dataset was analysed by descriptive statistics, correlation analysis, exploratory factor analysis (EFA), and hierarchical multiple linear regression. SPSS 24 was used to analyse the data. For further findings and interpretation, please refer to the research article entitled “*Corporate Governance in Extreme Institutional Environment: Evidence From Emerging Economy*” [1]. We also suggest referring to another article that was used to develop the survey questionnaire [2].

## Specifications Table

**Table:** 

Subject	Business, Management and Accounting
Specific subject area	Accounting and Finance
Type of data	The raw data is available in SPSS (sav) and excel formats while analysed data is presented in this paper.
How data were acquired	Self-administrated survey questionnaire. The survey questionnaire is also included in this paper.
Data format	Raw, analysed, descriptive and statistical data
Parameters for data collection	Five-point Likert scale ranging from strongly agree (1) to strongly disagree (5) and nominal scale.
Description of data collection	Purposive sampling method was applied. Firstly, only those PSX listed firms were selected which had recent annual reports available (2017/2018). Secondly, a survey questionnaire in English language was distributed to those auditors, accountants, managers or other members of PSX listed firms who participated in the preparation of CG reports. The survey questionnaire was distributed to 350 respondents from 350 PSX listed firms, nevertheless, 120 respondents returned the questionnaire. After reviewing the filled questionnaires, fifteen questionnaires were found to be incomplete. Therefore, the final sample consisted of 105 respondents from 105 PSX listed firms. Secondary data was collected from annual reports of respondents' firms. It took about six to eight weeks to conduct the survey.
Data source location	Pakistan
Data accessibility	Data are included in this paper.
Related research article	Arslan, M., Abidin, S., Alqatan, A., & Roudaki, J. (2019). Corporate Governance in Extreme Institutional Environment: Evidence from Emerging Economy. *Corporate Ownership & Control*, *17*(1). [1]

## Value of the Data

• The dataset presents the opinions of managers, auditors and accountants of PSX listed firms.
• The dataset provides a significant contribution in exploring the CG practices, their barriers and drivers.
• The dataset can be useful for managers, researchers, policymakers and regulators who are dealing with corporate governance issues and trying to find ways to improve CG practices.
• The dataset can be used to examine the relationship of CG compliance, barriers and drivers with other variables by employing different statistical techniques.

## 1 Data Description

Both primary and secondary data were collected. The dataset consists of following four parts and was collected through self-administrated survey questionnaire^1^:

1. The first part of the questionnaire is comprised of questions that helped in measuring the CG compliance among PSX listed firms. The five-point Likert scale ranging from (1) strongly disagree to (5) strongly agree was used. This part consisted of 48 provisions from Code of Corporate Governance 2012 and distributed into seven sub sections i.e. Auditing (7 items), Board of Directors (BoDs) (16 items), Charters/laws (8 items), Directors’ Education (4 items), Executive Director Compensation (7 items), Ownership (2 items), and Progressive Practice (4 items). CG index (CGI score) was computed by using this section which was used as independent variable in this study. Higher value of CGI score means high level of CG compliance in the firm and vice versa.
2. The second part of the questionnaire is comprised of of barriers to good CG practices in Pakistan, measured similarly to first part through a five-point Likert scale. This part has a total of 17 items. The full list of items can be seen in Appendix A. The 10 items such as BA1; BA2; BA4; BA5; BA6; BA10; BA11; BA13; BA16; and BA17 have eigen value of higher than 1 while the rest 7 items such as BA3; BA7; BA8; BA9; BA12; BA14; and BA15 have eigen values of less than 1^2^.
3. The third part of the questionnaire is comprised of drivers of good CG practices in Pakistan, measured similarly to first part through a five-point Likert scale. This part has a total of 12 items. The full list of items can be seen in Appendix A. The 7 items such as DR1; DR4; DR5; DR6; DR7; DR8; and DR10 have eigen value of greater than 1 while rest 5 items such as DR2; DR3; DR9; DR11; and DR12 have eigen value of less than 1^3^.
4. The last part of the questionnaire is comprised of respondents’ demographic information.

Secondary data were also collected from most recent annual reports (2017/2018) of the PSX listed firms^4^. The data were collected through self-administrated questionnaire due to several reasons such as time savings, ease of distribution, authenticity, ensure anonymity, and increase the response rate. The data are available in excel and SPSS formats with this paper. In addition, the results are also provided with the dataset both in excel and word files. Dataset file contains all primary and secondary data and is ready to use format. The questions are labelled same as showing in the survey questionnaire (see Appendix A in supplementary file). The future researchers can use full dataset or some parts of the dataset.

## 2 Experimental Design, Materials and Methods

The survey questionnaire was developed following the study of Arslan and Alqatan [2]. To ensure the face and content validity, the survey questionnaire was pre-tested by examining consistency and interpretation. In addition, superfluous and complex terms were eradicated, and naive words and language were used to ensure validity. The survey questionnaire was also sent for pilot testing to ensure validity. The data was collected from 15 respondents and items' reliability was checked through Cronbach alpha. The Cronbach alpha reveals the internal consistency and how closely related a set of items are as a group. It ranges from zero to one, however it is considered highly reliable if it has value above 0.70. Cronbach alphas for all three parts of questionnaire were above 0.70^5^, hence questionnaire was suitable to conduct the study.

In addition, data were analysed through descriptive statistics, correlation, exploratory factor analysis (EFA), and hierarchical multiple linear regression analysis with the help of SPSS24.

Firstly, descriptive statistics and correlation analysis were performed to get an overview of the data and identify the relationship among variables. Secondly, the EFA, an interdependence method, was performed to classify the most imperative barriers and drivers of good CG practices in Pakistan. The EFA reduces the items, having similar meanings, into smaller groups by investigating the inter-correlation between items. It provides the opportunity to researchers to identify fundamental dimensions and factors that exist in a given data set and is very expedient in research in lessening the items into discrete dimensions. At the end, hierarchical multiple linear regression model was estimated for the study, using below equation:

$$Firm\;Performance:\;\beta_{0}\;+\beta_{1}Demographic\;Variables\;+\;\beta_{2}CGI\;Score\;+\varepsilon$$

where

$\beta_{0}$ = Constant

Demographic variables: Part four of the survey questionnaire

Firm Performance: ROA and ROE from annual reports

CGI Score: Part one of the survey questionnaire

ε: Error term

## Ethics Statement

This research study was approved by the Human Ethics Committee of Lincoln University, New Zealand on 4th April 2017 with application No. 2017-18 under the project titled "Corporate Governance, Compliance and Performance Nexus". All the participants gave their informed consent^6^.

## Credit Authors Statement

Conceptualization – M.A.; Data Curation – M.A. and A.A.; Formal Analysis – M.A.; Investigation – M.A.; Methodology – M.A.; Project Administration – M.A. and A.A.; Resources – M.A. and A.A.; Validation – M.A.; Writing - Original Draft– M.A.; Writing –Review and Editing –M.A. and A.A.; Funding Acquisition – M.A. and A.A.

## Declaration of Competing Interest

The authors declare that they have no known competing financial interests or personal relationships which have, or could be perceived to have, influenced the work reported in this article.

## Appendix Supplementary materials

Image, application 1

## Footnotes

1 Survey questionnaire is available as Appendix A in supplementary file.

4 Data was collected only for those PSX listed firms, which participated in the survey questionnaire.

5 The results are not reported here. The full results can be found in the study of Arslan, M., et al., [1].

6 Sample consent form is available as Appendix B in supplementary file.

## Footnotes

2 See Appendix A part two in supplementary file.

3 See Appendix A part three in supplementary file.
