# Supplementary material for: Corporate governance practices, barriers and drivers: A survey dataset
Source: Data Brief. 2020 Nov 29;33:106603. doi: 10.1016/j.dib.2020.106603 (PMC7721603; doi:10.1016/j.dib.2020.106603)
Supplement: Supplementary file 1 [file mmc1.zip › Appendix A Survey Questionnaire.docx]

**Appendix A. Supplementary data**

# Survey Questionnaire

**Part One: Level of Corporate Governance Compliance**

Please indicate the level of your agreement on the compliance of each of the provisions of the Code of Corporate Governance 2012 (CCG) within your organization. Please circle or tick your answer.

**1 = Strongly Disagree, 2 = Disagree, 3 = Uncertain, 4 = Agree, 5 = Strongly Agree**

1. **Audit**

| 1. The Audit Committee should consist solely of independent non-executive outside directors. | **1** | **2** | **3** | **4** | **5** |
| --- | --- | --- | --- | --- | --- |
| 1. The Audit Committee of a listed company shall meet at least once every quarter of the financial year. | **1** | **2** | **3** | **4** | **5** |
| 1. The company should have a formal policy on external auditor rotation. | **1** | **2** | **3** | **4** | **5** |
| 1. The Chairman of the Audit Committee shall be an independent director, who shall not be the chairman of the board. | **1** | **2** | **3** | **4** | **5** |
| 1. The secretary of the Audit Committee shall either be the Company Secretary or Head of Internal Audit. | **1** | **2** | **3** | **4** | **5** |
| 1. A Human Resources and Remuneration committee should be introduced. | **1** | **2** | **3** | **4** | **5** |
| 1. The internal audit function may be outsourced by a listed company to a professional services firm or be performed by the internal audit staff of the holding company. | **1** | **2** | **3** | **4** | **5** |

1. **Board of Directors (BoDs)**

| 1. No former CEO should serve on the board. | **1** | **2** | **3** | **4** | **5** |
| --- | --- | --- | --- | --- | --- |
| 1. BoDs should meet at least once every quarter of the year. | **1** | **2** | **3** | **4** | **5** |
| 1. The board of directors of each listed company shall have at least one and preferably one third of the total members of the board as independent directors. | **1** | **2** | **3** | **4** | **5** |
| 1. Executive directors, i.e. working or whole-time directors, should not be more than 75% of the elected directors including the Chief Executive | **1** | **2** | **3** | **4** | **5** |
| 1. All directors should attend board meetings or have a valid excuse for non-attendance. | **1** | **2** | **3** | **4** | **5** |
| 1. As per CCG 2012, a director can serve on the board of seven listed companies at the most at any one time. | **1** | **2** | **3** | **4** | **5** |
| 1. Board members should be elected annually. | **1** | **2** | **3** | **4** | **5** |
| 1. Shareholder approval should be compulsory to change board size. | **1** | **2** | **3** | **4** | **5** |
| 1. Any casual vacancy on the board of directors of a listed company shall be filled up by the directors at the earliest but not later than 90 days. | **1** | **2** | **3** | **4** | **5** |
| 1. All written notices shall be circulated at least seven days prior to the meetings, except in the case of emergency. | **1** | **2** | **3** | **4** | **5** |
| 1. The Board of Directors shall clearly define the respective roles and responsibilities of the Chairman. | **1** | **2** | **3** | **4** | **5** |
| 1. The Board of Directors shall clearly define the respective roles and responsibilities of the CEO. | **1** | **2** | **3** | **4** | **5** |
| 1. The Chairman shall ensure that the minutes of meetings of the board of directors are appropriately recorded. | **1** | **2** | **3** | **4** | **5** |
| 1. The Chairman and CEO shall not be the same person. | **1** | **2** | **3** | **4** | **5** |
| 1. The Chairman shall be elected from amongst the non-executive directors of the listed company. | **1** | **2** | **3** | **4** | **5** |
| 1. There should a mechanism for undertaking annual evaluations of the performance of the Board. | **1** | **2** | **3** | **4** | **5** |

1. **Charter /Bylaws**

| 1. Shareholders should act by a non-unanimous written consent. | **1** | **2** | **3** | **4** | **5** |
| --- | --- | --- | --- | --- | --- |
| 1. Shareholders should be allowed to call special meetings. | **1** | **2** | **3** | **4** | **5** |
| 1. A simple majority but not a supermajority vote should be required to amend charter/bylaws. | **1** | **2** | **3** | **4** | **5** |
| 1. A simple majority but not a supermajority vote should be enough to approve a merger. | **1** | **2** | **3** | **4** | **5** |
| 1. The company should not be authorized to issue blank check preferred stock. | **1** | **2** | **3** | **4** | **5** |
| 1. The Board should not have right to amend bylaws without shareholder approval except under limited circumstances. | **1** | **2** | **3** | **4** | **5** |
| 1. Shareholders should have the right to vote on directors’ appointments to fill vacancies. | **1** | **2** | **3** | **4** | **5** |
| 1. Shareholders should have cumulative voting rights to elect directors. | **1** | **2** | **3** | **4** | **5** |

1. **Directors’ Education**

| 1. The CFO should have a post graduate qualification in accounting and finance. | **1** | **2** | **3** | **4** | **5** |
| --- | --- | --- | --- | --- | --- |
| 1. The CFO should have five years of experience of handling the financial or corporate affairs of a listed company or a bank or a financial institution. | **1** | **2** | **3** | **4** | **5** |
| 1. The company should encourage board members to attend professional training programs. | **1** | **2** | **3** | **4** | **5** |
| 1. It will be mandatory for directors of listed companies to attain certification under any SECP approved director training program (DTP). | **1** | **2** | **3** | **4** | **5** |

1. **Executive and Directors’ Compensation**

| 1. Directors’ remuneration packages shall encourage value creation within the company and be subject to prior approval of shareholders/board as required by the company’s Articles of Association. | **1** | **2** | **3** | **4** | **5** |
| --- | --- | --- | --- | --- | --- |
| 1. The appointment, removal, remuneration and terms and conditions of employment of the CFO of listed companies shall be determined by the Board. | **1** | **2** | **3** | **4** | **5** |
| 1. The appointment, removal, remuneration and terms and conditions of employment of the CS listed companies shall be determined by the Board. | **1** | **2** | **3** | **4** | **5** |
| 1. The appointment, remuneration and terms and conditions of employment of the Head of Internal Audit (IA) of listed companies shall be determined by the Board. | **1** | **2** | **3** | **4** | **5** |
| 1. There shall be a formal and transparent procedure for fixing the remuneration packages of individual directors. | **1** | **2** | **3** | **4** | **5** |
| 1. No director shall be involved in deciding his/her own remuneration. | **1** | **2** | **3** | **4** | **5** |
| 1. The company's Annual Report shall contain details of the aggregate remuneration separately of executive and non-executive directors, including salary/fee, benefits and performance-linked incentives etc. | **1** | **2** | **3** | **4** | **5** |

1. **Ownership**

| 1. If any director, CEO or executive of a listed company or their spouses sell, buy or transact, whether directly or indirectly, in shares of the listed company of which he is a director, CEO or executive, as the case may be, he shall immediately notify in writing to the Company Secretary of such transaction. | **1** | **2** | **3** | **4** | **5** |
| --- | --- | --- | --- | --- | --- |
| 1. Each listed company shall determine a closed period prior to the announcement of interim/ final result and no director, CEO or executive shall, directly or indirectly, deal in the shares of the listed company in any manner during the closed period. | **1** | **2** | **3** | **4** | **5** |

1. **Progressive Practices**

| 1. A board-approved CEO succession plan should be in place. | **1** | **2** | **3** | **4** | **5** |
| --- | --- | --- | --- | --- | --- |
| 1. The Board should have independent outside advisors. | **1** | **2** | **3** | **4** | **5** |
| 1. The Board should include independent directors. | **1** | **2** | **3** | **4** | **5** |
| 1. Director term limits should exist. | **1** | **2** | **3** | **4** | **5** |

**Part Two: Barriers to good corporate governance practices in Pakistan Stock Exchange (PSX) listed firms**

Please indicate the extent of your agreement as to whether each of the possible barriers below negatively impact the CG practices in Pakistan. Please circle or tick your answer.

**1 = Strongly Disagree, 2 = Disagree, 3 = Uncertain, 4 = Agree, 5 = Strongly Agree**

| 1. Lack of Auditors’ independence | **1** | **2** | **3** | **4** | **5** |
| --- | --- | --- | --- | --- | --- |
| 1. Board ineffectiveness | **1** | **2** | **3** | **4** | **5** |
| 1. Institutional Culture of Pakistan | **1** | **2** | **3** | **4** | **5** |
| 1. Political and Governmental interference in business activities | **1** | **2** | **3** | **4** | **5** |
| 1. Weak legal control and enforcement | **1** | **2** | **3** | **4** | **5** |
| 1. Lack of Shareholders’ awareness | **1** | **2** | **3** | **4** | **5** |
| 1. Lack of resources for CG compliance | **1** | **2** | **3** | **4** | **5** |
| 1. Lack of Shareholders’ rights protection especially minority shareholders | **1** | **2** | **3** | **4** | **5** |
| 1. Lack of protection for Whistle blowers | **1** | **2** | **3** | **4** | **5** |
| 1. Lack of professional education and training among stakeholders | **1** | **2** | **3** | **4** | **5** |
| 1. Fewer voting rights | **1** | **2** | **3** | **4** | **5** |
| 1. Low AGM participation | **1** | **2** | **3** | **4** | **5** |
| 1. High level of corruption | **1** | **2** | **3** | **4** | **5** |
| 1. Nepotism or Kinship culture | **1** | **2** | **3** | **4** | **5** |
| 1. Wobbly/unstable economy of Pakistan | **1** | **2** | **3** | **4** | **5** |
| 1. Strong social ties among different stakeholders | **1** | **2** | **3** | **4** | **5** |
| 1. Interpersonal connections among BoDs | **1** | **2** | **3** | **4** | **5** |

**Part Three: Drivers of good corporate governance practices in PSX listed firms**

Pease indicate the extent of your agreement as to whether each of the possible drivers below positively impacts the CG practices in Pakistan. Please circle or tick your answer.

**1 = Strongly Disagree, 2 = Disagree, 3 = Uncertain, 4 = Agree, 5 = Strongly Agree**

| 1. Auditors’ independence | **1** | **2** | **3** | **4** | **5** |
| --- | --- | --- | --- | --- | --- |
| 1. Internal control and Risk Management | **1** | **2** | **3** | **4** | **5** |
| 1. Provide protection to whistle blowers | **1** | **2** | **3** | **4** | **5** |
| 1. Enhancing and empowering professional regulatory bodies | **1** | **2** | **3** | **4** | **5** |
| 1. Board heterogeneity | **1** | **2** | **3** | **4** | **5** |
| 1. Board independence | **1** | **2** | **3** | **4** | **5** |
| 1. Encouraging participation in events and conferences related to corporate governance) | **1** | **2** | **3** | **4** | **5** |
| 1. Enhancing partnership with international regulatory bodies i.e. OECD, IFC to promote CG in Pakistan | **1** | **2** | **3** | **4** | **5** |
| 1. Provide accounting and auditing education to internal stakeholders | **1** | **2** | **3** | **4** | **5** |
| 1. Initiation of training programs for directors, raise awareness and education for CEOs, directors, shareholders and board members | **1** | **2** | **3** | **4** | **5** |
| 1. Establish corporate governance education programs at universities | **1** | **2** | **3** | **4** | **5** |
| 1. Promote CG research in Pakistan | **1** | **2** | **3** | **4** | **5** |

**Part Four: Demographic information**

Please place a tick in the appropriate box in each of these questions

1. **Age group**
2. 30 years or less
3. 31 to 40 years
4. 41 to 50 years
5. 51 to 60 years
6. Above 60 years
7. **Position within Organization**
8. Senior Manager

1. Manager
2. Accountant
3. Auditor
4. Other
5. **Qualifications**

1. PhD or Equivalent

1. Masters or equivalent
2. Bachelors or equivalent
3. Diploma or professional
4. Others
5. **Specialization of your latest education**
6. Finance

1. Accounting
2. Economics
3. Management
4. Others
5. **Experience within job**
6. 5 years or less

1. 6 to 10 years
2. 11 to 15 years
3. 16 to 20 years
4. Above 20 years

Thank you once again for your time and efforts in completing this questionnaire. If you have any comments, please mention them below:

__________________________________________________________________________________

_____________________________________________________________________________
