# Supplementary material for: Corporate governance practices, barriers and drivers: A survey dataset
Source: Data Brief. 2020 Nov 29;33:106603. doi: 10.1016/j.dib.2020.106603 (PMC7721603; doi:10.1016/j.dib.2020.106603)
Supplement: Supplementary file 1 [file mmc1.zip › EFA Barriers.doc]

FACTOR
  /VARIABLES BA1 BA2 BA3 BA4 BA5 BA6 BA7 BA8 BA9 BA10 BA11 BA12 BA13 BA14 BA15 BA16 BA17
  /MISSING LISTWISE
  /ANALYSIS BA1 BA2 BA3 BA4 BA5 BA6 BA7 BA8 BA9 BA10 BA11 BA12 BA13 BA14 BA15 BA16 BA17
  /PRINT UNIVARIATE INITIAL CORRELATION SIG DET KMO AIC EXTRACTION ROTATION
  /FORMAT BLANK(.5)
  /PLOT EIGEN
  /CRITERIA MINEIGEN(1) ITERATE(25)
  /EXTRACTION PAF
  /CRITERIA ITERATE(25)
  /ROTATION VARIMAX
  /METHOD=CORRELATION.


Factor Analysis


Descriptive Statistics	
	Mean	Std. Deviation	Analysis N	
BA1	3.5810	1.24639	105	
BA2	3.6762	1.34089	105	
BA3	3.7714	1.15407	105	
BA4	3.8381	1.10178	105	
BA5	3.7048	1.17607	105	
BA6	3.7714	1.28045	105	
BA7	3.3143	1.04066	105	
BA8	3.5238	1.09277	105	
BA9	2.9619	1.05542	105	
BA10	3.7810	.79640	105	
BA11	3.2381	.94588	105	
BA12	3.1048	.80770	105	
BA13	3.3524	1.02826	105	
BA14	3.1238	.71650	105	
BA15	3.0762	.70295	105	
BA16	3.1333	1.01968	105	
BA17	3.2857	.98756	105	


Correlation Matrixa	
	BA1	BA2	BA3	BA4	BA5	BA6	BA7	BA8										
Correlation	BA1	1.000	.626	.307	.517	.079	.614	.347	-.014										
	BA2	.626	1.000	.772	.543	.201	.830	.804	.340										
	BA3	.307	.772	1.000	.674	.495	.589	.685	.607										
	BA4	.517	.543	.674	1.000	.571	.485	.322	.423										
	BA5	.079	.201	.495	.571	1.000	.102	.336	.398										
	BA6	.614	.830	.589	.485	.102	1.000	.762	.217										
	BA7	.347	.804	.685	.322	.336	.762	1.000	.344										
	BA8	-.014	.340	.607	.423	.398	.217	.344	1.000										
	BA9	.463	.582	.435	.458	.185	.392	.300	.151										
	BA10	.052	.419	.322	.156	.300	.460	.699	.221										
	BA11	.183	.531	.244	-.064	-.334	.482	.392	.194										
	BA12	.512	.582	.181	.214	-.129	.498	.407	-.270										
	BA13	.334	.146	.158	.594	.500	.076	.003	.108										
	BA14	.048	-.398	-.338	-.303	-.264	-.357	-.388	-.292										
	BA15	.223	.190	.282	.227	.214	.148	.164	.348										
	BA16	-.319	.018	.190	-.109	.194	-.043	.078	.437										
	BA17	-.206	.172	.294	.061	.231	.197	.389	.466										
Sig. (1-tailed)	BA1		.000	.001	.000	.212	.000	.000	.445										
	BA2	.000		.000	.000	.020	.000	.000	.000										
	BA3	.001	.000		.000	.000	.000	.000	.000										
	BA4	.000	.000	.000		.000	.000	.000	.000										
	BA5	.212	.020	.000	.000		.151	.000	.000										
	BA6	.000	.000	.000	.000	.151		.000	.013										
	BA7	.000	.000	.000	.000	.000	.000		.000										
	BA8	.445	.000	.000	.000	.000	.013	.000											
	BA9	.000	.000	.000	.000	.030	.000	.001	.062										
	BA10	.299	.000	.000	.056	.001	.000	.000	.012										
	BA11	.031	.000	.006	.258	.000	.000	.000	.023										
	BA12	.000	.000	.033	.014	.095	.000	.000	.003										
	BA13	.000	.068	.054	.000	.000	.219	.487	.136										
	BA14	.314	.000	.000	.001	.003	.000	.000	.001										
	BA15	.011	.026	.002	.010	.014	.066	.047	.000										
	BA16	.000	.428	.026	.134	.024	.333	.215	.000										
	BA17	.017	.040	.001	.270	.009	.022	.000	.000										


KMO and Bartlett's Test	
Kaiser-Meyer-Olkin Measure of Sampling Adequacy.	.702	
Bartlett's Test of Sphericity	Approx. Chi-Square	1561.297	
	df	136	
	Sig.	.000	


Anti-image Matrices	
	BA1	BA2	BA3	BA4	BA5	BA6	BA7	BA8										
Anti-image Covariance	BA1	.179	-.050	.067	-.029	-.044	-.053	.005	-.010										
	BA2	-.050	.055	-.035	-.002	.018	-.006	-.025	-.009										
	BA3	.067	-.035	.100	-.057	-.034	.009	-.023	-.043										
	BA4	-.029	-.002	-.057	.108	-.033	-.055	.045	-.028										
	BA5	-.044	.018	-.034	-.033	.214	.064	-.044	.052										
	BA6	-.053	-.006	.009	-.055	.064	.161	-.048	.050										
	BA7	.005	-.025	-.023	.045	-.044	-.048	.084	.003										
	BA8	-.010	-.009	-.043	-.028	.052	.050	.003	.298										
	BA9	-.020	-.026	-.019	.011	.017	.028	-.005	.015										
	BA10	.034	.020	.009	.024	-.038	-.027	-.063	-.067										
	BA11	-.019	-.015	-.007	.034	.084	-.027	.024	-.054										
	BA12	.023	-.049	.024	-.019	.035	.026	-.006	.106										
	BA13	-.011	.013	.047	-.071	-.054	.021	-.003	-.038										
	BA14	-.131	.037	-.059	.035	.067	.042	-.016	.013										
	BA15	-.033	.008	-.007	.012	-.034	-.007	-.027	-.109										
	BA16	.054	-.013	-.015	.074	-.090	-.054	.032	-.051										
	BA17	.020	-.013	.049	-.069	.000	.027	-.045	-.030										
Anti-image Correlation	BA1	.649a	-.507	.497	-.207	-.225	-.312	.039	-.042										
	BA2	-.507	.796a	-.472	-.021	.163	-.065	-.374	-.069										
	BA3	.497	-.472	.712a	-.544	-.231	.074	-.247	-.246										
	BA4	-.207	-.021	-.544	.628a	-.217	-.420	.476	-.158										
	BA5	-.225	.163	-.231	-.217	.640a	.348	-.327	.204										
	BA6	-.312	-.065	.074	-.420	.348	.799a	-.417	.226										
	BA7	.039	-.374	-.247	.476	-.327	-.417	.751a	.020										
	BA8	-.042	-.069	-.246	-.158	.204	.226	.020	.753a										
	BA9	-.094	-.222	-.121	.069	.073	.141	-.037	.056										
	BA10	.167	.180	.062	.154	-.170	-.138	-.453	-.254										
	BA11	-.084	-.118	-.041	.194	.341	-.126	.156	-.186										
	BA12	.118	-.446	.164	-.124	.162	.137	-.045	.412										
	BA13	-.063	.126	.339	-.500	-.270	.122	-.022	-.162										
	BA14	-.537	.271	-.320	.186	.249	.183	-.093	.042										
	BA15	-.091	.040	-.027	.044	-.087	-.022	-.109	-.234										
	BA16	.244	-.103	-.091	.428	-.370	-.257	.208	-.179										
	BA17	.102	-.125	.341	-.459	.002	.147	-.336	-.122										


Communalities	
	Initial	Extraction	
BA1	.821	.790	
BA2	.945	.961	
BA3	.900	.768	
BA4	.892	.773	
BA5	.786	.771	
BA6	.839	.794	
BA7	.916	.905	
BA8	.702	.671	
BA9	.750	.777	
BA10	.770	.783	
BA11	.717	.835	
BA12	.780	.737	
BA13	.811	.765	
BA14	.664	.585	
BA15	.281	.153	
BA16	.722	.546	
BA17	.792	.751	

Extraction Method: Principal Axis Factoring.	


Total Variance Explained	
Factor	Initial Eigenvalues	Extraction Sums of Squared Loadings	Rotation Sums of Squared Loadings			
	Total	% of Variance	Cumulative %	Total	% of Variance	Cumulative %	Total			
1	5.861	34.476	34.476	5.661	33.299	33.299	3.671			
2	2.922	17.187	51.663	2.629	15.467	48.766	2.736			
3	2.279	13.408	65.072	2.050	12.060	60.826	2.541			
4	1.406	8.273	73.344	1.096	6.446	67.272	1.849			
5	1.182	6.954	80.298	.928	5.457	72.728	1.567			
6	.867	5.097	85.396							
7	.563	3.314	88.710							
8	.455	2.677	91.387							
9	.388	2.285	93.672							
10	.285	1.675	95.346							
11	.197	1.156	96.503							
12	.183	1.074	97.576							
13	.152	.895	98.471							
14	.096	.565	99.036							
15	.084	.496	99.532							
16	.042	.249	99.781							
17	.037	.219	100.000							


Factor Matrixa	
	Factor	
	1	2	3	4	5	
BA1	.570					
BA2	.933					
BA3	.792					
BA4	.679		.528			
BA5			.662			
BA6	.810					
BA7	.818					
BA8		.593				
BA9	.637					
BA10	.562			-.588		
BA11			-.650			
BA12	.517	-.595				
BA13			.639			
BA14	-.520					
BA15						
BA16		.677				
BA17		.817				

Extraction Method: Principal Axis Factoring.a	
a. 5 factors extracted. 13 iterations required.	


Rotated Factor Matrixa	
	Factor	
	1	2	3	4	5	
BA1	.748					
BA2	.866					
BA3	.580					
BA4		.762				
BA5		.752				
BA6	.822					
BA7	.698			.597		
BA8			.660			
BA9					.626	
BA10				.845		
BA11					.718	
BA12	.511		-.553			
BA13		.759				
BA14				-.530		
BA15						
BA16			.722			
BA17			.786			

Extraction Method: Principal Axis Factoring. 
 Rotation Method: Varimax with Kaiser Normalization.a	
a. Rotation converged in 12 iterations.	


Factor Transformation Matrix	
Factor	1	2	3	4	5	
1	.736	.440	.085	.391	.325	
2	-.117	-.018	.961	.177	-.176	
3	-.318	.892	-.040	-.171	-.270	
4	.512	-.024	.178	-.818	-.190	
5	.286	-.104	-.189	.342	-.869	

Extraction Method: Principal Axis Factoring.  
 Rotation Method: Varimax with Kaiser Normalization.	
