# Supplementary material for: Corporate governance practices, barriers and drivers: A survey dataset
Source: Data Brief. 2020 Nov 29;33:106603. doi: 10.1016/j.dib.2020.106603 (PMC7721603; doi:10.1016/j.dib.2020.106603)
Supplement: Supplementary file 1 [file mmc1.zip › EFA Drivers.doc]

FACTOR
  /VARIABLES DR1 DR2 DR3 DR4 DR5 DR6 DR7 DR8 DR9 DR10 DR11 DR12
  /MISSING LISTWISE
  /ANALYSIS DR1 DR2 DR3 DR4 DR5 DR6 DR7 DR8 DR9 DR10 DR11 DR12
  /PRINT UNIVARIATE INITIAL CORRELATION SIG DET KMO AIC EXTRACTION ROTATION
  /FORMAT BLANK(.5)
  /PLOT EIGEN
  /CRITERIA MINEIGEN(1) ITERATE(25)
  /EXTRACTION PC
  /CRITERIA ITERATE(25)
  /ROTATION VARIMAX
  /METHOD=CORRELATION.


Factor Analysis

Descriptive Statistics	
	Mean	Std. Deviation	Analysis N	
DR1	3.6762	.91457	105	
DR2	3.0667	.98319	105	
DR3	3.6571	.86412	105	
DR4	3.3524	.84331	105	
DR5	3.2762	.89330	105	
DR6	3.3714	.97327	105	
DR7	3.3810	.90278	105	
DR8	3.1810	.99789	105	
DR9	3.2667	.84656	105	
DR10	3.6476	.83183	105	
DR11	3.1238	.71650	105	
DR12	3.3238	.94559	105	


Correlation Matrixa	
	DR1	DR2	DR3	DR4	DR5	DR6	DR7	DR8					
Correlation	DR1	1.000	.559	.588	.349	.817	.709	.209	.075					
	DR2	.559	1.000	.661	.563	.669	.667	.426	-.071					
	DR3	.588	.661	1.000	.669	.547	.610	.132	.006					
	DR4	.349	.563	.669	1.000	.418	.343	.176	-.077					
	DR5	.817	.669	.547	.418	1.000	.677	.214	.008					
	DR6	.709	.667	.610	.343	.677	1.000	.155	-.020					
	DR7	.209	.426	.132	.176	.214	.155	1.000	-.024					
	DR8	.075	-.071	.006	-.077	.008	-.020	-.024	1.000					
	DR9	.038	.129	-.176	-.052	.080	.112	.117	-.251					
	DR10	.809	.382	.459	.069	.663	.638	.129	.031					
	DR11	.531	.370	.659	.468	.622	.499	-.014	.103					
	DR12	.456	.432	.667	.278	.451	.704	.046	.060					
Sig. (1-tailed)	DR1		.000	.000	.000	.000	.000	.016	.222					
	DR2	.000		.000	.000	.000	.000	.000	.235					
	DR3	.000	.000		.000	.000	.000	.090	.477					
	DR4	.000	.000	.000		.000	.000	.037	.219					
	DR5	.000	.000	.000	.000		.000	.014	.467					
	DR6	.000	.000	.000	.000	.000		.057	.418					
	DR7	.016	.000	.090	.037	.014	.057		.404					
	DR8	.222	.235	.477	.219	.467	.418	.404						
	DR9	.350	.096	.036	.299	.210	.128	.116	.005					
	DR10	.000	.000	.000	.242	.000	.000	.094	.376					
	DR11	.000	.000	.000	.000	.000	.000	.443	.148					
	DR12	.000	.000	.000	.002	.000	.000	.322	.273					


KMO and Bartlett's Test	
Kaiser-Meyer-Olkin Measure of Sampling Adequacy.	.735	
Bartlett's Test of Sphericity	Approx. Chi-Square	868.428	
	df	66	
	Sig.	.000	


Anti-image Matrices	
	DR1	DR2	DR3	DR4	DR5	DR6	DR7	DR8					
Anti-image Covariance	DR1	.145	.045	-.045	-.046	-.089	-.036	-.028	-.077					
	DR2	.045	.238	-.091	-.041	-.095	-.077	-.170	-.012					
	DR3	-.045	-.091	.201	-.110	.048	.029	.067	.027					
	DR4	-.046	-.041	-.110	.308	.018	-.031	-.044	.071					
	DR5	-.089	-.095	.048	.018	.184	-.018	.024	.033					
	DR6	-.036	-.077	.029	-.031	-.018	.218	.081	.014					
	DR7	-.028	-.170	.067	-.044	.024	.081	.724	-.018					
	DR8	-.077	-.012	.027	.071	.033	.014	-.018	.871					
	DR9	-.005	-.043	.096	-.046	-.031	-.023	.006	.175					
	DR10	-.103	-.001	-.015	.129	.024	-.038	-.037	.072					
	DR11	.051	.053	-.019	-.111	-.098	.054	.045	-.053					
	DR12	.019	.019	-.086	.095	.034	-.133	-.048	-.014					
Anti-image Correlation	DR1	.753a	.241	-.261	-.218	-.546	-.203	-.088	-.217					
	DR2	.241	.763a	-.418	-.153	-.453	-.338	-.410	-.026					
	DR3	-.261	-.418	.794a	-.443	.249	.140	.177	.065					
	DR4	-.218	-.153	-.443	.625a	.074	-.121	-.093	.137					
	DR5	-.546	-.453	.249	.074	.779a	-.089	.065	.083					
	DR6	-.203	-.338	.140	-.121	-.089	.817a	.205	.032					
	DR7	-.088	-.410	.177	-.093	.065	.205	.557a	-.023					
	DR8	-.217	-.026	.065	.137	.083	.032	-.023	.358a					
	DR9	-.017	-.102	.248	-.095	-.083	-.057	.008	.216					
	DR10	-.608	-.003	-.073	.519	.125	-.180	-.097	.172					
	DR11	.294	.240	-.091	-.440	-.501	.256	.116	-.125					
	DR12	.104	.079	-.398	.354	.162	-.591	-.118	-.030					


Communalities	
	Initial	Extraction	
DR1	1.000	.828	
DR2	1.000	.813	
DR3	1.000	.848	
DR4	1.000	.823	
DR5	1.000	.770	
DR6	1.000	.766	
DR7	1.000	.771	
DR8	1.000	.751	
DR9	1.000	.682	
DR10	1.000	.842	
DR11	1.000	.772	
DR12	1.000	.662	

Extraction Method: Principal Component Analysis.	


Total Variance Explained	
Component	Initial Eigenvalues	Extraction Sums of Squared Loadings	Rotation Sums of Squared Loadings			
	Total	% of Variance	Cumulative %	Total	% of Variance	Cumulative %	Total			
1	5.504	45.866	45.866	5.504	45.866	45.866	4.228			
2	1.545	12.872	58.738	1.545	12.872	58.738	2.404			
3	1.252	10.437	69.174	1.252	10.437	69.174	1.354			
4	1.026	8.554	77.728	1.026	8.554	77.728	1.340			
5	.701	5.842	83.571							
6	.667	5.554	89.125							
7	.459	3.824	92.949							
8	.307	2.556	95.505							
9	.224	1.869	97.375							
10	.155	1.289	98.663							
11	.091	.761	99.424							
12	.069	.576	100.000							


Component Matrixa	
	Component	
	1	2	3	4	
DR1	.845				
DR2	.769				
DR3	.831				
DR4	.574		-.694		
DR5	.849				
DR6	.844				
DR7		.535		.627	
DR8		-.530		.675	
DR9		.738			
DR10	.742		.534		
DR11	.764				
DR12	.738				

Extraction Method: Principal Component Analysis.a	
a. 4 components extracted.	


Rotated Component Matrixa	
	Component	
	1	2	3	4	
DR1	.867				
DR2		.554	.500		
DR3	.513	.758			
DR4		.880			
DR5	.792				
DR6	.804				
DR7			.869		
DR8				.824	
DR9				-.733	
DR10	.915				
DR11	.636	.509			
DR12	.658				

Extraction Method: Principal Component Analysis. 
 Rotation Method: Varimax with Kaiser Normalization.a	
a. Rotation converged in 6 iterations.	


Component Transformation Matrix	
Component	1	2	3	4	
1	.836	.522	.164	.018	
2	-.066	-.063	.626	-.774	
3	.543	-.825	-.131	-.085	
4	-.033	-.205	.751	.627	

Extraction Method: Principal Component Analysis.  
 Rotation Method: Varimax with Kaiser Normalization.	
