# Supplementary material for: Corporate governance practices, barriers and drivers: A survey dataset
Source: Data Brief. 2020 Nov 29;33:106603. doi: 10.1016/j.dib.2020.106603 (PMC7721603; doi:10.1016/j.dib.2020.106603)
Supplement: Supplementary file 1 [file mmc1.zip › Regression ROE.doc]

REGRESSION
  /MISSING LISTWISE
  /STATISTICS COEFF OUTS R ANOVA COLLIN TOL
  /CRITERIA=PIN(.05) POUT(.10)
  /NOORIGIN
  /DEPENDENT ROE
  /METHOD=ENTER Age Position Qual Specialisation Exp
  /METHOD=ENTER Total_CGI.


Regression


Variables Entered/Removeda	
Model	Variables Entered	Variables Removed	Method	
1	Exp, Qual, Specialisation, Position, Ageb	.	Enter	
2	Total_CGIb	.	Enter	

a. Dependent Variable: ROE	
b. All requested variables entered.	


Model Summary	
Model	R	R Square	Adjusted R Square	Std. Error of the Estimate	
1	.256a	.065	.018	.177906	
2	.474b	.225	.178	.162824	

a. Predictors: (Constant), Exp, Qual, Specialisation, Position, Age	
b. Predictors: (Constant), Exp, Qual, Specialisation, Position, Age, Total_CGI	


ANOVAa	
Model	Sum of Squares	df	Mean Square	F	Sig.	
1	Regression	.219	5	.044	1.386	.236b	
	Residual	3.133	99	.032			
	Total	3.353	104				
2	Regression	.755	6	.126	4.744	.000c	
	Residual	2.598	98	.027			
	Total	3.353	104				

a. Dependent Variable: ROE	
b. Predictors: (Constant), Exp, Qual, Specialisation, Position, Age	
c. Predictors: (Constant), Exp, Qual, Specialisation, Position, Age, Total_CGI	


Coefficientsa	
Model	Unstandardized Coefficients	Standardized Coefficients	t	Sig.	Collinearity Statistics	
	B	Std. Error	Beta			Tolerance	VIF	
1	(Constant)	.069	.098		.702	.484			
	Age	.038	.030	.209	1.298	.197	.364	2.751	
	Position	-.003	.020	-.017	-.171	.865	.903	1.108	
	Qual	.026	.021	.125	1.193	.236	.857	1.166	
	Specialisation	.021	.019	.121	1.135	.259	.831	1.203	
	Exp	-.069	.038	-.291	-1.842	.068	.378	2.648	
2	(Constant)	-.803	.214		-3.760	.000			
	Age	.020	.027	.107	.714	.477	.355	2.817	
	Position	.012	.019	.059	.617	.539	.874	1.144	
	Qual	.023	.020	.110	1.148	.254	.856	1.168	
	Specialisation	.009	.017	.050	.502	.617	.810	1.235	
	Exp	-.027	.036	-.116	-.771	.443	.352	2.842	
	Total_CGI	.005	.001	.431	4.493	.000	.861	1.161	

a. Dependent Variable: ROE	


Excluded Variablesa	
Model	Beta In	t	Sig.	Partial Correlation	Collinearity Statistics	
					Tolerance	VIF	Minimum Tolerance	
1	Total_CGI	.431b	4.493	.000	.413	.861	1.161	.352	

a. Dependent Variable: ROE	
b. Predictors in the Model: (Constant), Exp, Qual, Specialisation, Position, Age	


Collinearity Diagnosticsa	
Model	Dimension	Eigenvalue	Condition Index	Variance Proportions			
				(Constant)	Age	Position	Qual	Specialisation			
1	1	5.455	1.000	.00	.00	.00	.00	.00			
	2	.236	4.807	.00	.02	.26	.07	.18			
	3	.158	5.877	.00	.06	.08	.00	.42			
	4	.098	7.467	.01	.02	.56	.54	.01			
	5	.035	12.477	.40	.40	.03	.24	.09			
	6	.019	17.136	.59	.50	.06	.15	.29			
2	1	6.424	1.000	.00	.00	.00	.00	.00			
	2	.236	5.216	.00	.02	.26	.07	.18			
	3	.161	6.325	.00	.06	.05	.00	.36			
	4	.103	7.907	.00	.03	.60	.39	.00			
	5	.050	11.298	.03	.09	.01	.49	.29			
	6	.023	16.644	.00	.76	.01	.03	.16			
	7	.003	44.558	.97	.05	.07	.01	.00			
