# Supplementary material for: Corporate governance practices, barriers and drivers: A survey dataset
Source: Data Brief. 2020 Nov 29;33:106603. doi: 10.1016/j.dib.2020.106603 (PMC7721603; doi:10.1016/j.dib.2020.106603)
Supplement: Supplementary file 1 [file mmc1.zip › Appendix B Consent Form for Participants.docx]

**Consent Form for Participants**

**Project Title:** Corporate Governance, Compliance and Performance Nexus

**Research Student:** (Deleted to keep anonymity)

**Project Supervisory Team:** (Deleted to keep anonymity)

- I have read and understood the description of the project entitled **“Corporate Governance, Compliance and Performance Nexus”**.
- I understand the procedures explained in the information sheet and I give my consent to participate in the project.
- I consent to publication of the data and results of the project with the understanding that anonymity will be preserved.
- I understand that I may withdraw my participation from the project at any stage until 30^th^ June 2017 and that this withdrawal will not jeopardise me in any way.
- I understand that information relevant to me will be deleted in case of my withdrawal.
- I have been informed that the information I provide will be used only for research purpose and anonymity will be kept.
- Would you like to receive copy of the findings of study Yes / No

**Participant Signature:** _______________________ **Date:_____________________**

**Participant Name (Optional):** ____________________________

**Researcher Contact Details:**

*Please send signed copy to researcher at (deleted to keep anonymity)
